# Cryptococcosis After Initiation of Janus Kinase Inhibitors or Biologic DMARDs in Rheumatoid Arthritis: Three Pairwise Active-Comparator Analyses of Propensity Score-Matched TriNetX Cohorts, 2005–2025

**DOI:** 10.3390/ph19101650

**Published:** 2026-10-19

**Authors:** Ying Xiu Tay, Yi-Ting He, Malcolm Koo, Ming-Chi Lu

**Affiliations:** 1 Division of Allergy, Immunology and Rheumatology, Dalin Tzu Chi Hospital, Buddhist Tzu Chi Medical Foundation, Dalin 622401, Chiayi, Taiwan; 2 Center for Health Data & Advanced Translational Analytics, Dalin Tzu Chi Hospital, Buddhist Tzu Chi Medical Foundation, Dalin 622401, Chiayi, Taiwan; 3 Department of Public Health, College of Medicine, National Cheng Kung University, Tainan City 704302, Taiwan; 4 Department of Medical Research, Dalin Tzu Chi Hospital, Buddhist Tzu Chi Medical Foundation, Dalin 622401, Chiayi, Taiwan; 5 Dalla Lana School of Public Health, University of Toronto, Toronto, ON M5T 3M7, Canada; 6 School of Medicine, Tzu Chi University, Hualien City 970374, Hualien, Taiwan

**Keywords:** rheumatoid arthritis, Janus kinase inhibitors, cryptococcosis, opportunistic infection, TriNetX, real-world evidence, pharmacovigilance, post-authorization safety

## Abstract

**Background/Objectives:** Comparative safety evidence for cryptococcosis during advanced rheumatoid arthritis (RA) therapy is limited. We compared newly recorded cryptococcosis after initiation of Janus kinase inhibitors (JAKi), tumor necrosis factor (TNF) inhibitors (TNFi), and non-TNF biologic disease-modifying antirheumatic drugs (non-TNF bDMARDs). **Methods:** Three retrospective active-comparator analyses were conducted in the TriNetX Global Collaborative Network: JAKi versus TNFi, JAKi versus non-TNF bDMARDs, and TNFi versus non-TNF bDMARDs. After 1:1 propensity score matching, outcomes from day 1 to 10 years after initiation, capped at 30 June 2025, were analyzed using Cox proportional hazards models. E-values assessed sensitivity to unmeasured confounding. **Results:** Matched cohorts included 27,833, 32,440, and 37,351 patients per group, respectively; all absolute standardized mean differences were <0.10. Events occurred in 20 JAKi and 12 TNFi initiators (hazard ratio [HR], 2.20; 95% confidence interval [CI], 1.06–4.54) and in 22 JAKi and 13 non-TNF bDMARD initiators (HR, 2.15; 95% CI, 1.07–4.31). In the secondary comparison, 17 TNFi and 19 non-TNF bDMARD initiators had events (HR, 0.83; 95% CI, 0.43–1.60). Point-estimate E-values for the JAKi comparisons were 3.82 and 3.72; CI-limit E-values were 1.31 and 1.34. **Conclusions:** JAKi initiation was associated with an approximately twofold higher hazard of newly recorded cryptococcosis than either biologic comparator, while estimated 10-year cumulative event probabilities were below 0.2% in all groups. The separately selected and matched populations do not support an indirect ranking of treatment classes. Low event counts, wide CIs, possible outcome misclassification, fixed exposure classification, and residual confounding preclude causal conclusions. The findings require independent confirmation.

## 1. Introduction

Rheumatoid arthritis (RA) is a chronic systemic autoimmune disease in which genetic susceptibility, environmental triggers, and autoantibody production contribute to persistent inflammation and joint damage [1]. An estimated 17.6 million people are affected worldwide, and prevalence is projected to rise [2]. Sustained immunomodulatory treatment is often required to control disease activity and prevent structural damage [3]. Therapies include conventional synthetic disease-modifying antirheumatic drugs (DMARDs), biologic DMARDs (bDMARDs), and targeted synthetic DMARDs such as Janus kinase inhibitors (JAKi) [4]. Comparative safety is clinically important because modulation of immune pathways can increase susceptibility to serious and opportunistic infections [5].

Cryptococcosis is an invasive fungal infection caused primarily by *Cryptococcus neoformans* and *Cryptococcus gattii*. Pulmonary and central nervous system involvement are the most recognized manifestations, although disseminated infection can affect multiple organs and be life-threatening, particularly in immunocompromised individuals [6]. Patients with RA may be susceptible because of disease-related immune dysregulation and immunosuppressive treatment [7]. In a Taiwanese retrospective case–control study of 9132 patients with newly diagnosed RA, cryptococcosis occurred in 0.22%. All affected patients had received corticosteroids; chronic kidney disease and adalimumab use were independently associated with increased risk [8].

JAKi provide an effective oral treatment option for RA, but serious and opportunistic infections remain important safety concerns. In the Oral Rheumatoid Arthritis Trial (ORAL) Surveillance study, serious infection events were more frequent with tofacitinib than with tumor necrosis factor (TNF) inhibitors (TNFi), particularly at 10 mg twice daily; increasing age, chronic lung disease, and oral corticosteroid use were important risk factors [9]. Opportunistic infections, including fungal infections, were also reported in the tofacitinib RA clinical development program [10], and serious infections remained a concern in post-marketing surveillance [11]. Cryptococcosis-specific evidence is sparse: pulmonary cryptococcosis during baricitinib or other JAKi therapy and disseminated infection during tofacitinib treatment have been described [12,13,14]. These cases demonstrate occurrence during treatment but cannot establish comparative risk.

Broader opportunistic-infection outcomes cannot establish cryptococcosis-specific comparative risk because aggregate estimates may be driven by more frequent infections. Cryptococcosis is rare, and conventional trials and single-center cohorts generally provide too few events for stable comparative estimates. Large real-world datasets can extend post-authorization safety evaluation, but disease characteristics, comorbidities, previous therapies, and infection risk factors influence RA treatment selection and create potential confounding.

To address this gap, we used the TriNetX Global Collaborative Network [15] to construct three separate pairwise propensity score-matched active-comparator cohorts. The two primary comparisons evaluated JAKi versus TNFi and JAKi versus non-TNF bDMARDs; the secondary comparison evaluated TNFi versus non-TNF bDMARDs. We estimated comparator-specific associations with newly recorded cryptococcosis after treatment initiation. The separately selected and matched cohorts were not intended to support an indirect treatment ranking or a class-wide causal conclusion.

## 2. Results

### 2.1. Matched Cohorts and Baseline Balance

**Figure 1:**
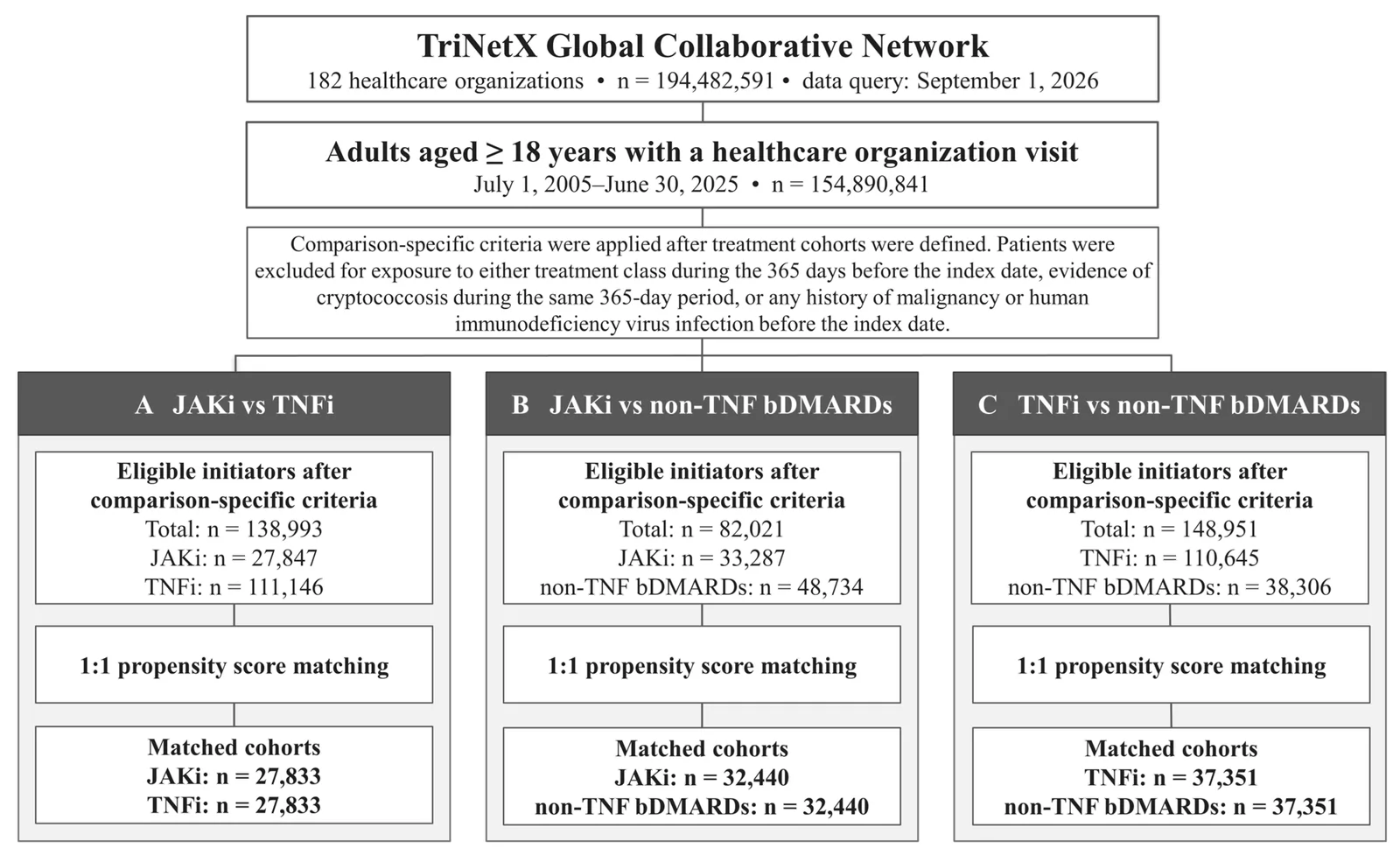
Cohort selection and propensity score matching for three pairwise treatment comparisons among patients with rheumatoid arthritis: (A) JAKi versus TNFi; (B) JAKi versus non-TNF bDMARDs; and (C) TNFi versus non-TNF bDMARDs. Eligible initiators were identified after application of comparison-specific criteria, and propensity score matching was performed separately for each comparison at a 1:1 ratio. Exposure to either treatment class being compared and evidence of cryptococcosis during the 365 days before the index date were exclusion criteria. Malignant neoplasm and HIV infection were assessed at any time before the index date. bDMARDs, biologic disease-modifying antirheumatic drugs; HIV, human immunodeficiency virus; JAKi, Janus kinase inhibitor; RA, rheumatoid arthritis; TNF, tumor necrosis factor; TNFi, tumor necrosis factor inhibitor.

After 1:1 propensity score matching, the JAKi versus TNFi comparison included 27,833 patients per group, the JAKi versus non-TNF bDMARDs comparison included 32,440 patients per group, and the TNFi versus non-TNF bDMARDs comparison included 37,351 patients per group (Figure 1).

**Figure 2:**
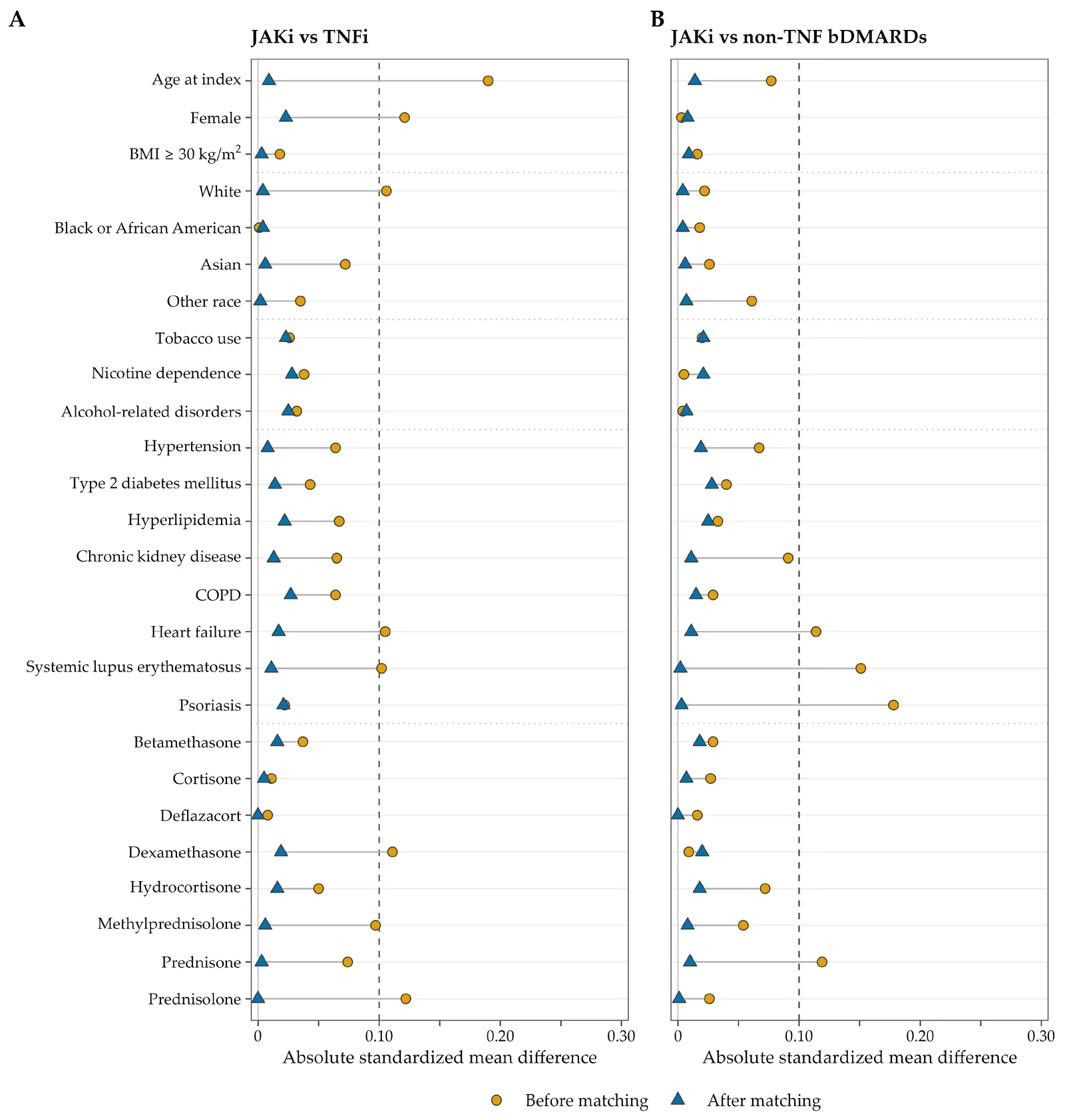
Covariate balance before and after propensity score matching for the two primary treatment comparisons: (**A**) JAKi versus TNFi and (**B**) JAKi versus non-TNF bDMARDs. Orange circles and blue triangles represent absolute standardized mean differences before and after matching, respectively. The vertical dashed line at 0.10 indicates the prespecified threshold for acceptable covariate balance. BMI, body mass index; bDMARDs, biologic disease-modifying antirheumatic drugs; COPD, chronic obstructive pulmonary disease; JAKi, Janus kinase inhibitor; TNF, tumor necrosis factor; TNFi, tumor necrosis factor inhibitor.

Propensity score matching reduced measured baseline differences. Absolute standardized mean differences (SMDs) before and after matching were in the range of 0.001–0.190 and 0.000–0.028, respectively, for JAKi versus TNFi; 0.003–0.178 and 0.000–0.028 for JAKi versus non-TNF bDMARDs; and 0.006–0.262 and 0.001–0.034 for TNFi versus non-TNF bDMARDs. All post-matching absolute SMDs were below 0.10. Complete baseline characteristics and covariate-specific pre- and post-matching SMDs are provided in Supplementary Tables S2, S3 and S4, respectively. Covariate balance is also displayed in Figure 2 and Supplementary Figure S1.

### 2.2. Cryptococcosis Events and Relative Hazards

In the matched JAKi-versus-TNFi cohorts, 20 and 12 patients, respectively, had newly recorded cryptococcosis (Table 1). The estimated hazard ratio (HR) was 2.20 (95% confidence interval [CI] 1.06–4.54; log-rank *p* = 0.030). In the JAKi-versus-non-TNF bDMARD cohorts, 22 and 13 patients, respectively, had newly recorded cryptococcosis (HR 2.15, 95% CI 1.07–4.31; log-rank *p* = 0.028). The corresponding point-estimate E-values were 3.82 and 3.72, and CI-limit E-values were 1.31 and 1.34. In the secondary TNFi-versus-non-TNF bDMARD comparison, 17 and 19 events occurred, respectively (HR 0.83, 95% CI 0.43–1.60; log-rank *p* = 0.585). The proportional hazards assumption was not rejected in any comparison (all *p* ≥ 0.195). Each HR applies to its own selected and matched population; the estimates do not support an indirect ranking of treatment classes.

### 2.3. Cumulative Event Probability

**Figure 3:**
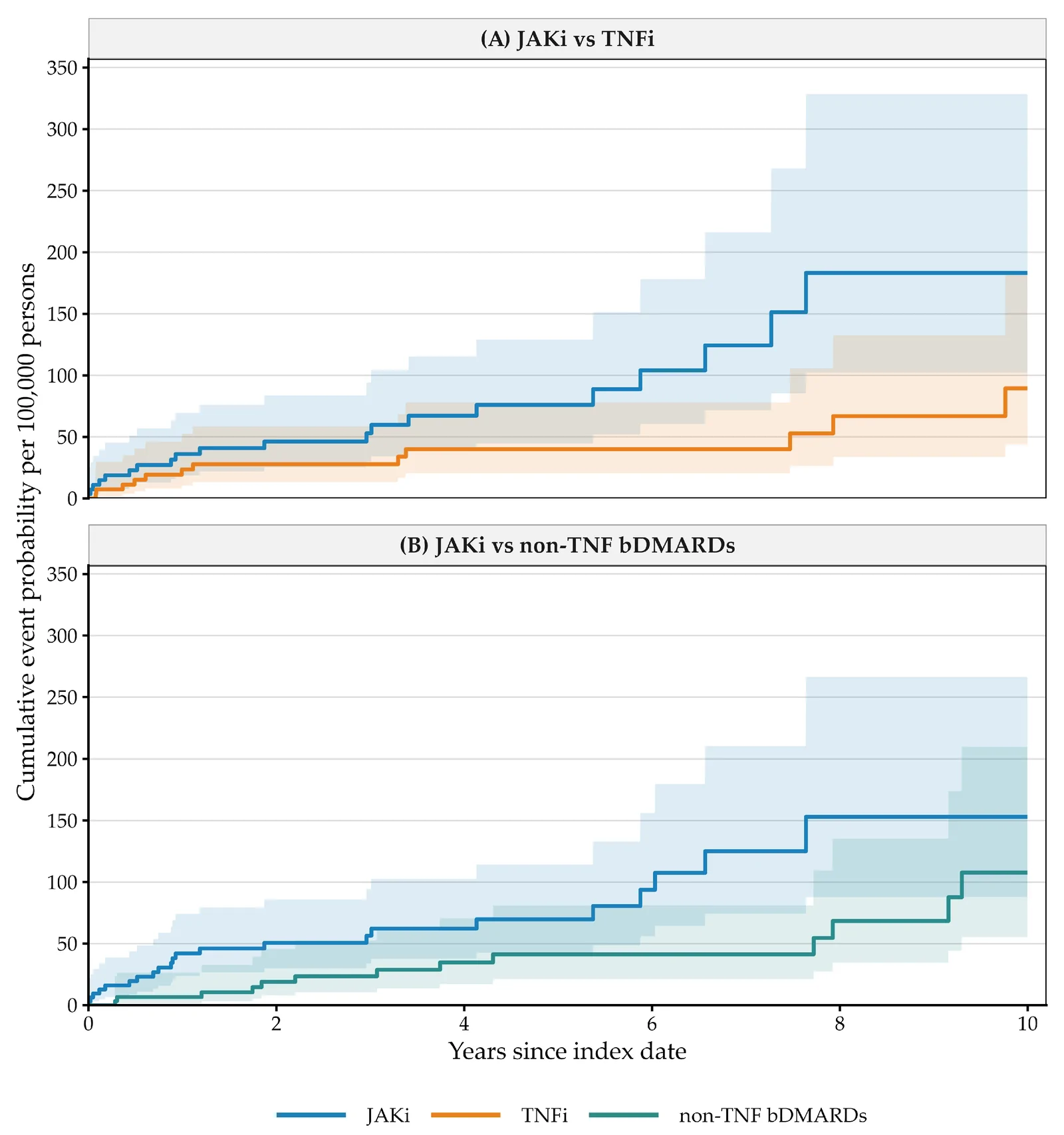
Cumulative event probability of cryptococcosis for the two primary treatment comparisons among patients with rheumatoid arthritis: (**A**) JAKi versus TNFi and (**B**) JAKi versus non-TNF bDMARDs. Cumulative event probability was calculated as 1 − *S*(*t*), where *S*(*t*) denotes the Kaplan–Meier estimate of cryptococcosis-free survival. Estimates are expressed per 100,000 persons. Solid step lines represent the estimated cumulative event probabilities, and shaded areas represent 95% confidence intervals. bDMARDs, biologic disease-modifying antirheumatic drugs; JAKi, Janus kinase inhibitor; TNF, tumor necrosis factor; TNFi, tumor necrosis factor inhibitor.

The estimated cumulative event probability of newly recorded cryptococcosis was higher in the JAKi cohort than in either comparator cohort (Figure 3A,B). At 10 years, estimates were 183 versus 89 per 100,000 persons for JAKi and TNFi, respectively, and 153 versus 108 per 100,000 persons for JAKi and non-TNF bDMARDs, respectively. In the secondary comparison, the curves remained relatively close, with estimated 10-year probabilities of 84 and 103 per 100,000 persons for TNFi and non-TNF bDMARDs, respectively (Supplementary Figure S2).

Because follow-up was capped at 30 June 2025, only earlier entrants with sufficient observation could contribute to the 10-year estimates; these do not imply complete 10-year follow-up for all participants. Confidence intervals widened later in follow-up as events remained sparse and observation declined, limiting interpretation of the long-term estimates.

## 3. Discussion

JAKi initiation was associated with an approximately twofold higher hazard of newly recorded cryptococcosis than either biologic comparator in the primary matched analyses. The secondary biologic comparison was inconclusive. Relative hazards should be considered alongside the low estimated 10-year cumulative event probabilities: 183 versus 89 per 100,000 persons for JAKi versus TNFi and 153 versus 108 per 100,000 persons for JAKi versus non-TNF bDMARDs. These estimates were below 0.2% in all groups but remained uncertain, particularly late in follow-up, and should not be interpreted as precise individual risk predictions.

Previous evidence linking JAKi therapy to cryptococcosis consisted largely of individual cases and small observational reports [12,13,14]. A review of cryptococcosis during biologic and targeted therapy described treatment-related immune effects and concurrent immunosuppression [16]. The French Research Axed on Tolerance of Biotherapies (RATIO) registry documented opportunistic infections, including invasive fungal infections, during TNF inhibition [17], and a structured review identified opportunistic infections as an established JAKi safety concern [18].

Broader comparative studies provide infection-safety context. A Korean national insurance cohort of RA patients initiating JAKi or TNFi during 2009–2020 used propensity-score fine stratification and weighting to examine hospitalized viral, fungal, and tuberculous infections. The composite HR was 1.94 (95% CI 1.44–2.64), while the fungal-infection estimate was elevated but imprecise (HR 2.07, 95% CI 0.59–7.26) [19]. A Dutch prescription-database cohort examined adults starting tofacitinib, baricitinib, or subcutaneous bDMARDs during 2018–2021, using multilevel Poisson regression of treatment episodes and antimicrobial prescriptions as infection proxies [20]. Our study used multinational electronic health record (EHR) data, separate pairwise matching, and a cryptococcosis-specific diagnosis code or serum-antigen definition, with treatment fixed at entry. Differences in populations, exposures, ascertainment, and design limit direct comparison; broader infection outcomes do not establish cryptococcosis-specific risk or independently replicate our findings.

The near-null estimate in the secondary comparison does not establish therapeutic equivalence, given its few events and wide CI. Each HR represents an association within a separately selected and matched population, preventing an indirect treatment ranking. Moreover, non-TNF bDMARDs include agents with distinct mechanisms and infection profiles, so the estimate for this group averages across a heterogeneous treatment category.

Biological explanations remain hypothesis-generating. Host defense against *Cryptococcus* involves T-helper 1 responses, cytokine signaling, and macrophage activation [16]. Janus kinase inhibition may affect these pathways, with consequences varying by selectivity, dose, duration, and concomitant therapy. TNF signaling also contributes to antifungal defense, and cryptococcosis and other invasive fungal infections have been reported during TNF inhibition [8,17]. These observations provide mechanistic context. The relevant pathways were not evaluated here, so the study cannot distinguish pharmacologic effects from residual group differences or other sources of bias.

Under the rare-outcome approximation, point-estimate E-values were 3.82 and 3.72 for the two JAKi comparisons. These provide equal-strength benchmarks for hypothetical treatment–confounder and confounder–outcome associations on the risk-ratio scale, conditional on measured covariates [21]; they do not estimate the strength of confounders actually present. CI-limit E-values of 1.31 and 1.34 indicate that comparatively modest unmeasured confounding could shift the lower confidence limits to the null. The larger point-estimate values therefore do not establish freedom from residual confounding. E-values also do not address misclassification, selection bias, or informative censoring.

The findings may inform awareness of cryptococcosis during advanced RA therapy, although their clinical relevance requires independent confirmation. Cryptococcosis should remain in the differential diagnosis of compatible pulmonary, neurologic, or disseminated illness, particularly with glucocorticoid exposure, chronic kidney disease, or other impairment of cellular immunity. The study did not compare individual JAKi agents or doses and provides no evidence supporting routine antigen testing in asymptomatic patients or avoidance of JAKi therapy.

### Strengths and Limitations

The large multinational electronic health record network enabled study of a rare outcome across three active-comparator cohorts. Separate matching balanced measured covariates and combining diagnosis codes with positive serum antigen results broadened case ascertainment.

Several limitations warrant consideration. First, the primary comparisons included only 32 and 35 total events, and the secondary comparison included 36, resulting in wide confidence intervals and imprecise long-term estimates. Median follow-up, interquartile ranges, and time-specific numbers at risk were unavailable from the analysis outputs accessible to us, limiting assessment of support for the 10-year estimates. Patient overlap between the separately matched primary cohorts was also unknown, so their similar estimates should not be viewed as independent replication.

Second, propensity score matching balanced measured baseline covariates but could not resolve residual confounding. Detailed measures of RA activity, functional status, treatment line, previous treatment response, cumulative immunosuppression, and glucocorticoid dose and duration were unavailable or incompletely captured in the accessible data. RA disease duration and detailed prior DMARD treatment history were not included in the model. These characteristics may influence both treatment selection and infection susceptibility, creating potential confounding by indication and disease severity. Matching on baseline glucocorticoid exposure did not establish comparability in dose, duration, or cumulative exposure. Healthcare utilization, testing intensity, and geographic exposure to Cryptococcus were also absent from the model. The direction and magnitude of residual confounding cannot be determined.

Third, treatment class was assigned at index and was not updated for discontinuation, switching, or subsequent combination therapy. Later events may therefore have occurred after treatment changes, limiting their attribution to the index therapy. Electronic health record medication entries may also not confirm dispensing or adherence.

Fourth, outcomes were identified from diagnosis codes or positive serum cryptococcal antigen results without clinical adjudication. Information on infection site, severity, microbiologic confirmation, and treatment was inconsistently available, and differential testing may have affected ascertainment. Follow-up from day 1 could capture infections already present but undocumented at initiation despite exclusion of recorded cryptococcosis during the preceding 365 days. The outcome therefore represents newly recorded cryptococcosis and does not establish infection onset after treatment initiation.

Fifth, calendar time was not controlled. Because JAKi entered clinical practice later than TNFi, treatment groups may have differed in diagnostic practices, treatment sequencing, background care, and available follow-up. These calendar-period differences may have contributed to the observed associations.

Finally, reasons for observation ending were unavailable, preventing distinction between death and loss to follow-up; informative censoring cannot be excluded. Death was not modeled as a competing event, so Kaplan–Meier estimates may overestimate the probability of cryptococcosis occurring before death. This bias could not be quantified, and mortality differences between groups may affect comparisons. Together with sparse events and limited follow-up information, these limitations restrict interpretation of the 10-year estimates as absolute risks.

## 4. Materials and Methods

### 4.1. Data Source and Study Design

This retrospective cohort study used the TriNetX Global Collaborative Network (TriNetX, LLC, Cambridge, MA, USA), a federated electronic health record platform for real-world clinical research. The network provides de-identified demographic, diagnostic, procedure, laboratory, and medication data from more than 180 healthcare organizations [15]. The study period was 1 July 2005 to 30 June 2025; the latter date also capped outcome follow-up. The administrative cutoff was selected to comply with the study hospital’s ethics requirements governing the period eligible for retrospective medical-record review. Queries and analyses were performed on 1 September 2026, using records available at that time. No formal sample size calculation was performed; all eligible patients were included before matching. Reporting followed the REporting of studies Conducted using Observational Routinely collected health Data (RECORD) statement.

The Human Research Ethics Committee of Dalin Tzu Chi Hospital, Buddhist Tzu Chi Medical Foundation, approved the protocol (Approval No. B11404033; date of approval: 22 December 2025) and exempted this retrospective study from the requirement to obtain informed consent. The study used existing data de-identified in accordance with 45 Code of Federal Regulations (CFR) 164.514(a) of the Health Insurance Portability and Accountability Act (HIPAA) Privacy Rule and involved no intervention or interaction with participants.

### 4.2. Cohort Definitions and Treatment Comparisons

Patients with RA aged ≥18 years who initiated a qualifying treatment during the study period were eligible. The index date was the first qualifying exposure determining cohort membership. Patients with any prior malignancy or evidence of human immunodeficiency virus (HIV) infection, identified by an HIV diagnosis code or positive HIV test, were excluded. Patients with a cryptococcosis diagnosis or positive serum antigen result during the preceding 365 days were also excluded.

Treatment class at index determined membership in each JAKi, TNFi, or non-TNF bDMARD cohort and remained fixed throughout follow-up, regardless of discontinuation, switching, or subsequent combination therapy. Medications in each class are listed in Supplementary Table S1.

Three comparisons were evaluated: JAKi versus TNFi, JAKi versus non-TNF bDMARDs, and TNFi versus non-TNF bDMARDs. Each excluded patients with recorded exposure to either compared class during the 365 days before the index. The washout covered all agents within those two classes, as defined in Supplementary Table S1. The washout did not extend to the third advanced-treatment class, conventional synthetic DMARDs, or corticosteroids. Patients could have prior RA treatment experience, including exposure to a compared class outside the washout window; the index exposure was therefore not necessarily first-ever use. Figure 1 summarizes cohort selection and matching.

### 4.3. Outcome Definition and Follow-Up

The primary outcome was newly recorded cryptococcosis, defined by an International Classification of Diseases, Tenth Revision, Clinical Modification (ICD-10-CM) diagnosis code B45 or a positive serum cryptococcal antigen result identified by Logical Observation Identifiers Names and Codes (LOINC) 29903-2 (version 2.83; released on 19 August 2026) [22]. Combining diagnosis codes and laboratory evidence was intended to broaden ascertainment.

Follow-up began on day 1 after index and ended at the first qualifying cryptococcosis event, the last available follow-up date recorded in TriNetX, 10 years after index, or 30 June 2025, whichever occurred first. Patients whose follow-up ended without an event were censored. The available output did not distinguish loss to follow-up, death, or other reasons for observation ending.

### 4.4. Propensity Score Matching

Patients were matched 1:1 separately for each comparison using a greedy nearest-neighbor propensity score algorithm with a caliper of 0.1 pooled standard deviations, consistent with the TriNetX platform’s standard matching protocol. Covariates included age, sex, race, body mass index ≥ 30 kg/m^2^, tobacco use, nicotine dependence, alcohol-related disorders, hypertension, type 2 diabetes mellitus, hyperlipidemia, chronic kidney disease, chronic obstructive pulmonary disease, heart failure, systemic lupus erythematosus, psoriasis, and baseline exposure to betamethasone, cortisone, deflazacort, dexamethasone, hydrocortisone, methylprednisolone, prednisone, and prednisolone. These covariates were selected to capture measured patient characteristics potentially relevant to treatment selection and infection susceptibility. Post-matching covariate balance was evaluated using standardized mean differences (SMDs), with an absolute SMD < 0.10 considered indicative of acceptable balance.

### 4.5. Statistical Analysis

Kaplan–Meier methods estimated cryptococcosis-free survival, and log-rank tests compared treatment groups. Cumulative event probability was calculated as 1 − *S*(*t*), where *S*(*t*) denotes the Kaplan–Meier estimate of cryptococcosis-free survival. Estimates are expressed per 100,000 persons. Death was not modeled as a competing event; these estimates do not represent cumulative incidence accounting for competing mortality.

Cox proportional hazards models were fitted separately within each matched cohort to estimate HRs and 95% CIs. Each model included only a binary treatment-group indicator, with one regression coefficient and no post-matching covariate adjustment. TriNetX survival-analysis outputs were exported to R (version 4.5.3; R Foundation for Statistical Computing, Vienna, Austria) for additional calculation and visualization.

The proportional hazards assumption was evaluated using the Schoenfeld-residual test provided in the exported analysis. E-values were calculated for HR point estimates and the 95% confidence limits closest to the null [21]. Under the rare-outcome approximation, HRs were treated as approximate risk ratios. For ratios of at least 1, the E-value was calculated by adding the ratio to the square root of the product of the ratio and (the ratio minus 1); ratios below 1 were first inverted. CI-limit E-values were set to 1 when the confidence interval included the null.

Sensitivity analyses restricting follow-up to 1 and 2 years were attempted, but sparse outcome counts triggered TriNetX privacy suppression and prevented reporting of comparative estimates. A sensitivity analysis restricted to ICD-10-CM B45 diagnosis codes was also attempted but did not yield reportable comparative estimates.

All tests were two-sided. Effect estimates and 95% CIs were emphasized because events were rare. No formal multiplicity adjustment was applied; reported *p* values and CIs are nominal. Analyses used available TriNetX data without imputation or interpolation.

### 4.6. Use of Generative Artificial Intelligence

OpenAI ChatGPT (GPT-5.6 Sol) was used to assist with language editing, document formatting, and review of aggregate analysis outputs. It was not used to define study cohorts, specify or execute TriNetX queries, or independently conduct the statistical analyses. The authors reviewed and verified all generated content and take full responsibility for the manuscript.

## 5. Conclusions

JAKi initiation was associated with an approximately twofold higher hazard of newly recorded cryptococcosis than either biologic comparator. Estimated 10-year Kaplan–Meier cumulative event probabilities were below 0.2% in all groups but remained uncertain. The secondary biologic comparison was inconclusive. The separately selected and matched populations do not support an indirect treatment ranking. Low event counts, wide CIs, non-adjudicated outcome ascertainment, fixed exposure classification, and residual confounding preclude causal conclusions. These hypothesis-generating findings require independent replication and do not establish a basis for routine cryptococcal screening, avoidance of JAKi therapy, or changes in treatment policy.

## Figures and Tables

**Table 1:** Hazard ratios, E-values, and proportional hazards test results for cryptococcosis across rheumatoid arthritis treatment comparisons.

Comparison	Matched *n* per Group	Events	HR (95% CI)	Log-Rank *p* Value	E-Value (CI-Limit E-Value)	PH Test *p*
JAKi vs. TNFi	27,833	20 vs. 12	2.20 (1.06–4.54)	0.030	3.82 (1.31)	0.759
JAKi vs. non-TNF bDMARDs	32,440	22 vs. 13	2.15 (1.07–4.31)	0.028	3.72 (1.34)	0.195
TNFi vs. non-TNF bDMARDs	37,351	17 vs. 19	0.83 (0.43–1.60)	0.585	1.69 (1.00)	0.609

CI, confidence interval; bDMARDs, biologic disease-modifying antirheumatic drugs; HR, hazard ratio; JAKi, Janus kinase inhibitor; PH, proportional hazards; TNF, tumor necrosis factor; TNFi, tumor necrosis factor inhibitor. Each row represents a separate pairwise matched population. Event counts are presented in the same order as the treatment classes in the comparison column.

## Data Availability

The datasets presented in this article are not readily available due to privacy and contractual restrictions, the raw data cannot be shared publicly. Requests to access the datasets should be directed from TriNetX (https://trinetx.com) subject to institutional approval and licensing requirements.

## Supplementary Materials

The following supporting information can be downloaded at https://www.mdpi.com/article/10.3390/ph19101650/s1, Figure S1: Covariate balance before and after propensity score matching for TNFi versus non-TNF bDMARDs; Figure S2: Cumulative event probability of newly recorded cryptococcosis for TNFi versus non-TNF bDMARDs; Table S1: Code definitions used in the study; Table S2: Baseline characteristics before and after propensity score matching for JAKi versus TNFi; Table S3: Baseline characteristics before and after propensity score matching for JAKi versus non-TNF bDMARDs; Table S4: Baseline characteristics before and after propensity score matching for TNFi versus non-TNF bDMARDs.

## Abbreviations

The following abbreviations are used in the manuscript and Supplementary Materials:

bDMARDs	Biologic disease-modifying antirheumatic drugs
BMI	Body mass index
CI	Confidence interval
COPD	Chronic obstructive pulmonary disease
CFR	Code of Federal Regulations
DMARDs	Disease-modifying antirheumatic drugs
EHR	Electronic health record
HIPAA	Health Insurance Portability and Accountability Act
HIV	Human immunodeficiency virus
HR	Hazard ratio
ICD-10-CM	International Classification of Diseases, Tenth Revision, Clinical Modification
JAKi	Janus kinase inhibitor
LOINC	Logical Observation Identifiers Names and Codes
non-TNF bDMARDs	Non-tumor necrosis factor biologic disease-modifying antirheumatic drugs
OMOP	Observational Medical Outcomes Partnership
ORAL	Oral Rheumatoid Arthritis Trial
PH	Proportional hazards
RA	Rheumatoid arthritis
RATIO	Research Axed on Tolerance of Biotherapies
RECORD	REporting of studies Conducted using Observational Routinely collected health Data
SD	Standard deviation
SMD	Standardized mean difference
TNF	Tumor necrosis factor
TNFi	Tumor necrosis factor inhibitor
TNX	TriNetX
