# Supplementary material for: Cryptococcosis After Initiation of Janus Kinase Inhibitors or Biologic DMARDs in Rheumatoid Arthritis: Three Pairwise Active-Comparator Analyses of Propensity Score-Matched TriNetX Cohorts, 2005–2025
Source: Pharmaceuticals (Basel). 2026 Oct 19;19(10):1650. doi: 10.3390/ph19101650 (PMC13708528; doi:10.3390/ph19101650)
Supplement: Supplementary file 1 [file pharmaceuticals-19-01650-s001.zip › Supplementary Table S4.pdf]

**Supplementary Table S4.** Baseline characteristics before and after propensity score matching: TNFi versus non-TNF bDMARDs.

| Variable | Before matching | | | After matching | | |
|---|---|---|---|---|---|---|
| | **TNFi** (n = 110,645) | **Non-TNF bDMARDs** (n = 38,306) | **SMD** | **TNFi** (n = 37,351) | **Non-TNF bDMARDs** (n = 37,351) | **SMD** |
| **Mean age at index, years (SD)** | 54.00 (14.9) | 57.88 (14.7) | 0.262 | 58.31 (14.3) | 57.82 (14.6) | 0.034 |
| **Female** | 84,208 (76.1) | 30,889 (80.6) | 0.110 | 30,452 (81.5) | 30,034 (80.4) | 0.029 |
| **Body mass index ≥ 30 kg/m²** | 33,855 (30.6) | 10,902 (28.5) | 0.047 | 10,479 (28.1) | 10,579 (28.3) | 0.006 |
| **Race** | | | | | | |
| White | 75,616 (68.3) | 23,826 (62.2) | 0.129 | 23,592 (63.2) | 23,420 (62.7) | 0.010 |
| Black or African American | 13,091 (11.8) | 4,779 (12.5) | 0.020 | 4,463 (12.0) | 4,572 (12.2) | 0.009 |
| Asian | 3,782 (3.4) | 1,556 (4.1) | 0.034 | 1,450 (3.9) | 1,496 (4.0) | 0.006 |
| Other | 4,001 (3.6) | 1,530 (4.0) | 0.020 | 1,335 (3.6) | 1,472 (4.0) | 0.019 |
| **Risk factors** | | | | | | |
| Tobacco use | 1,990 (1.8) | 577 (1.5) | 0.023 | 474 (1.3) | 569 (1.5) | 0.022 |
| Nicotine dependence | 6,514 (5.9) | 1,934 (5.0) | 0.037 | 1,626 (4.4) | 1,882 (5.0) | 0.032 |
| Alcohol-related disorders | 1,257 (1.1) | 311 (0.8) | 0.033 | 251 (0.7) | 309 (0.8) | 0.018 |
| **Comorbidities** | | | | | | |
| Hypertension | 26,380 (23.8) | 11,241 (29.4) | 0.125 | 10,456 (28.0) | 10,685 (28.6) | 0.014 |
| Type 2 diabetes mellitus | 10,792 (9.8) | 4,686 (12.2) | 0.079 | 4,251 (11.4) | 4,446 (11.9) | 0.016 |
| Hyperlipidemia | 13,105 (11.8) | 5,707 (14.9) | 0.090 | 5,222 (14.0) | 5,400 (14.5) | 0.014 |
| Chronic kidney disease | 3,810 (3.4) | 2,689 (7.0) | 0.161 | 2,188 (5.9) | 2,353 (6.3) | 0.018 |
| COPD | 3,877 (3.5) | 2,018 (5.3) | 0.086 | 1,738 (4.6) | 1,882 (5.0) | 0.018 |
| Heart failure | 2,284 (2.1) | 2,411 (6.3) | 0.213 | 1,913 (5.1) | 1,997 (5.4) | 0.010 |
| Systemic lupus erythematosus | 1,888 (1.7) | 2,619 (6.8) | 0.256 | 1,777 (4.8) | 1,969 (5.3) | 0.024 |
| Psoriasis | 6,837 (6.2) | 983 (2.6) | 0.177 | 917 (2.5) | 978 (2.6) | 0.010 |
| **Medication** | | | | | | |
| Betamethasone | 2,601 (2.4) | 938 (2.4) | 0.006 | 786 (2.1) | 887 (2.4) | 0.018 |
| Cortisone | 105 (0.1) | 64 (0.2) | 0.020 | 61 (0.2) | 59 (0.2) | 0.001 |
| Deflazacort | 23 (0.02) | 30 (0.08) | 0.026 | 22 (0.06) | 26 (0.07) | 0.004 |
| Dexamethasone | 7,015 (6.3) | 3,500 (9.1) | 0.105 | 3,089 (8.3) | 3,235 (8.7) | 0.014 |
| Hydrocortisone | 3,076 (2.8) | 1,866 (4.9) | 0.109 | 1,508 (4.0) | 1,634 (4.4) | 0.017 |
| Methylprednisolone | 16,506 (14.9) | 7,562 (19.7) | 0.128 | 6,678 (17.9) | 7,099 (19.0) | 0.029 |
| Prednisone | 38,780 (35.0) | 13,866 (36.2) | 0.024 | 12,835 (34.4) | 13,348 (35.7) | 0.029 |
| Prednisolone | 3,312 (3.0) | 2,275 (5.9) | 0.143 | 2,104 (5.6) | 2,089 (5.6) | 0.002 |

COPD, chronic obstructive pulmonary disease; non-TNF bDMARDs, non-tumor necrosis factor biologic disease-modifying antirheumatic drugs; SD, standard deviation; SMD, standardized mean difference; TNFi, tumor necrosis factor inhibitor. Data are presented as number (percentage) unless otherwise indicated.
