# Supplementary material for: Cryptococcosis After Initiation of Janus Kinase Inhibitors or Biologic DMARDs in Rheumatoid Arthritis: Three Pairwise Active-Comparator Analyses of Propensity Score-Matched TriNetX Cohorts, 2005–2025
Source: Pharmaceuticals (Basel). 2026 Oct 19;19(10):1650. doi: 10.3390/ph19101650 (PMC13708528; doi:10.3390/ph19101650)
Supplement: Supplementary file 1 [file pharmaceuticals-19-01650-s001.zip › Supplementary Figure S1.pdf]

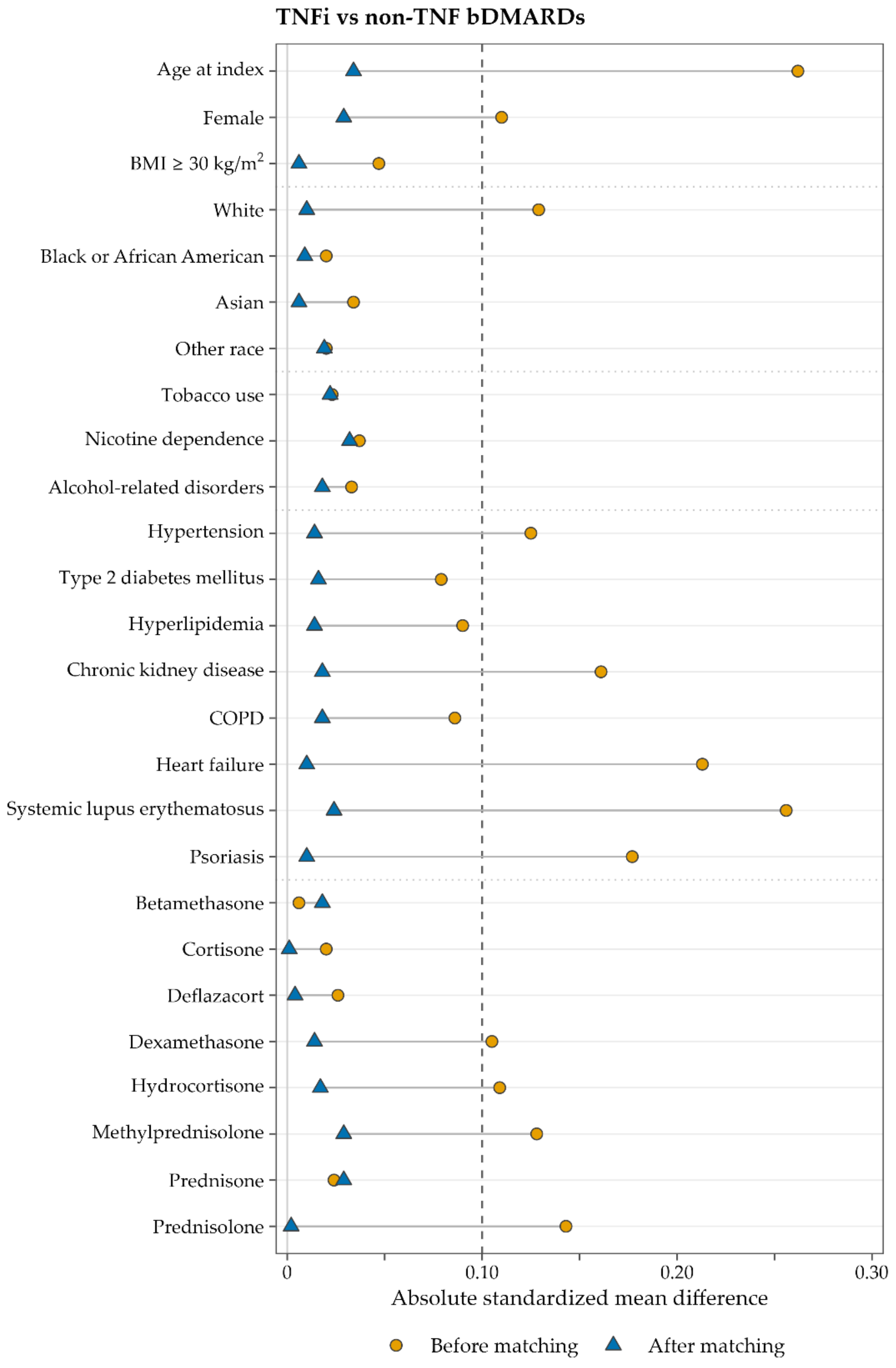

Figure S1. Covariate balance before and after propensity score matching for the secondary comparison of TNFi versus non-TNF bDMARDs. Orange circles and blue triangles represent absolute standardized mean differences before and after matching, respectively. The vertical dashed line at 0.10 indicates the prespecified threshold for acceptable balance. BMI, body mass index; COPD, chronic obstructive pulmonary disease; bDMARDs, biologic disease-modifying antirheumatic drugs; SMD, standardized mean difference; TNF, tumor necrosis factor; TNFi, tumor necrosis factor inhibitor.
