# Supplementary material for: Cryptococcosis After Initiation of Janus Kinase Inhibitors or Biologic DMARDs in Rheumatoid Arthritis: Three Pairwise Active-Comparator Analyses of Propensity Score-Matched TriNetX Cohorts, 2005–2025
Source: Pharmaceuticals (Basel). 2026 Oct 19;19(10):1650. doi: 10.3390/ph19101650 (PMC13708528; doi:10.3390/ph19101650)
Supplement: Supplementary file 1 [file pharmaceuticals-19-01650-s001.zip › Supplementary Figure S2.pdf]

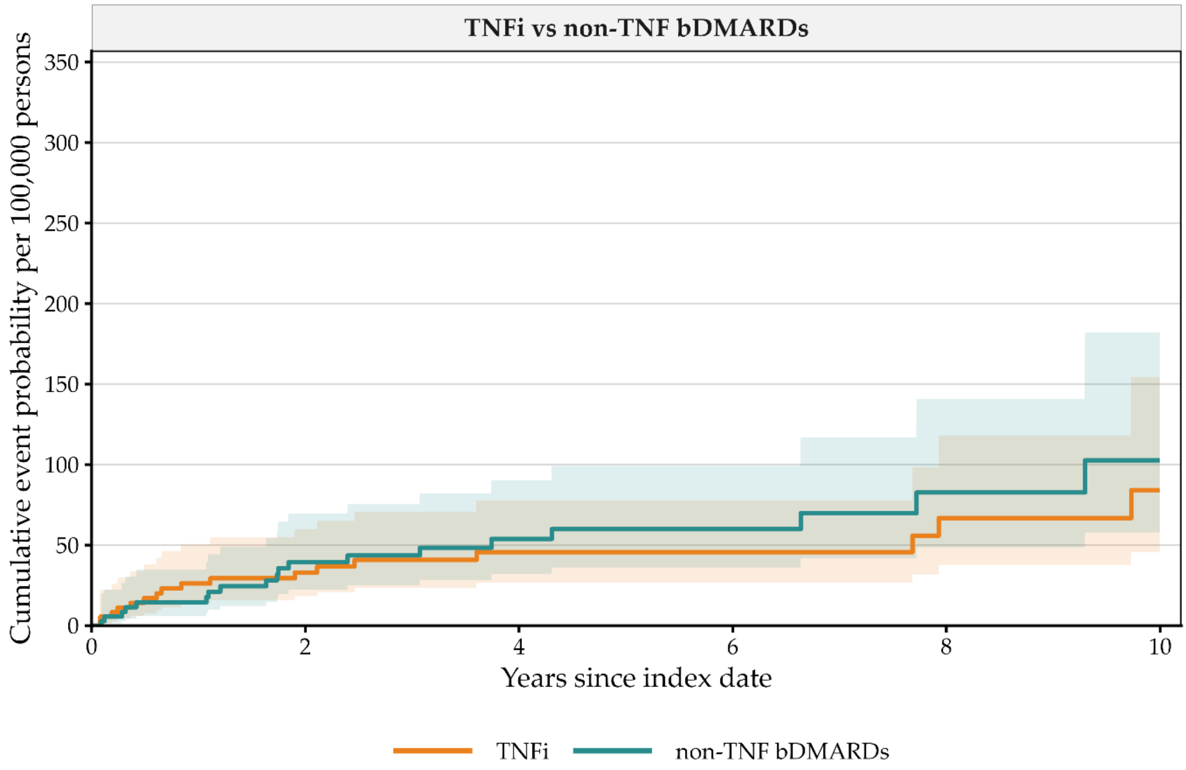

Figure S2. Cumulative event probability of newly recorded cryptococcosis after initiation of TNFi versus non-TNF bDMARDs. Cumulative event probability was calculated as $1 - S(t)$, where $S(t)$ denotes the Kaplan–Meier estimate of cryptococcosis-free survival. Estimates are expressed per 100,000 persons. Solid step lines show the estimated probabilities, and shaded areas show 95% confidence intervals. bDMARDs, biologic disease-modifying antirheumatic drugs; TNF, tumor necrosis factor; TNFi, tumor necrosis factor inhibitor.
