# Supplementary material for: Cryptococcosis After Initiation of Janus Kinase Inhibitors or Biologic DMARDs in Rheumatoid Arthritis: Three Pairwise Active-Comparator Analyses of Propensity Score-Matched TriNetX Cohorts, 2005–2025
Source: Pharmaceuticals (Basel). 2026 Oct 19;19(10):1650. doi: 10.3390/ph19101650 (PMC13708528; doi:10.3390/ph19101650)
Supplement: Supplementary file 1 [file pharmaceuticals-19-01650-s001.zip › Supplementary Table S1.pdf]

Supplementary Table S1. Code definitions used in the study.

Category		Study definition	Coding system	Codes
1. Population		Rheumatoid arthritis	ICD-10-CM	M05, M06
2. Study medication				
	Janus kinase inhibitor (JAKi)	Tofacitinib	RxNorm	1357536
		Baricitinib	RxNorm	2047232
		Upadacitinib	RxNorm	2196092
		Filgotinib	OMOP RxNorm Extension	OMOP5036197
	Tumor necrosis factor inhibitor biologic disease-modifying antirheumatic drug (TNFi)	Etanercept	RxNorm	214555
		Adalimumab	RxNorm	327361
		Golimumab	RxNorm	819300
		Certolizumab pegol	RxNorm	709271
		Infliximab	RxNorm	191831
	Non-tumor necrosis factor biologic disease-modifying antirheumatic drugs (non-TNF bDMARDs)	Sarilumab	RxNorm	1923319
		Tocilizumab	RxNorm	612865
		Abatacept	RxNorm	614391
		Rituximab	RxNorm	121191
3. Exclusion		Malignancy	ICD-10-CM	C00–C97
		HIV infection	ICD-10-CM	B20, Z21, B97.35
		Positive HIV test result	LOINC	56888-1, 7917-8, 10902-5

	Prior cryptococcosis	ICD-10-CM	B45
	Prior positive Cryptococcus species antigen test	LOINC	29903-2
4. Covariates	Age at index	TNX Demographic	AI
	Female	TNX Demographic	F
	White	TNX Demographic	2106-3
	Black or African American	TNX Demographic	2054-5
	Asian	TNX Demographic	2028-9
	Other race	TNX Demographic	2131-1
	Tobacco use	ICD-10-CM	Z72.0
	Nicotine dependence	ICD-10-CM	F17.2
	Alcohol-related disorders	ICD-10-CM	F10
	Essential (primary) hypertension	ICD-10-CM	I10
	Type 2 diabetes mellitus	ICD-10-CM	E11
	Hyperlipidemia, unspecified	ICD-10-CM	E78.5
	Chronic kidney disease	ICD-10-CM	N18
	Systemic lupus erythematosus	ICD-10-CM	M32
	Psoriasis	ICD-10-CM	L40
	Other chronic obstructive pulmonary disease	ICD-10-CM	J44
	Heart failure	ICD-10-CM	I50
	Body mass index	TNX Curated	9083
	Betamethasone	RxNorm	1514
	Cortisone	RxNorm	2878
	Deflazacort	RxNorm	22396
	Dexamethasone	RxNorm	3264
	Hydrocortisone	RxNorm	5492
	Methylprednisolone	RxNorm	6902
	Prednisolone	RxNorm	8638

	Prednisone	RxNorm	8640
5. Outcome	Cryptococcosis	ICD-10-CM	B45
	Cryptococcus species antigen test positive	LOINC	29903-2

HIV, human immunodeficiency virus; ICD-10-CM, International Classification of Diseases, Tenth Revision, Clinical Modification; LOINC, Logical Observation Identifiers Names and Codes; OMOP, Observational Medical Outcomes Partnership; OMOP RxNorm Extension, extension of RxNorm within the OMOP vocabulary used to represent medications not available in standard RxNorm; RxNorm, standardized clinical drug terminology developed by the U.S. National Library of Medicine; TNX, TriNetX.

TNX Curated indicates standardized clinical data and terminologies harmonized across the TriNetX network; TNX Demographic indicates demographic fields used in the TriNetX network. Medication concepts were identified using RxNorm or, when applicable, the OMOP RxNorm Extension.

For comparison-specific medication exclusions, patients were excluded if they had been exposed to either treatment class being compared during the 365 days before the index date. Prior cryptococcosis was assessed over the same 365-day period.
