# Supplementary material for: Cryptococcosis After Initiation of Janus Kinase Inhibitors or Biologic DMARDs in Rheumatoid Arthritis: Three Pairwise Active-Comparator Analyses of Propensity Score-Matched TriNetX Cohorts, 2005–2025
Source: Pharmaceuticals (Basel). 2026 Oct 19;19(10):1650. doi: 10.3390/ph19101650 (PMC13708528; doi:10.3390/ph19101650)
Supplement: Supplementary file 1 [file pharmaceuticals-19-01650-s001.zip › Supplementary Table S2.pdf]

Supplementary Table S2. Baseline characteristics of patients before and after propensity score matching: JAKi versus TNFi.

Variable	Before matching			After matching		
	JAKi (n = 27,847)	TNFi (n = 111,146)	SMD	JAKi (n = 27,833)	TNFi (n = 27,833)	SMD
**Mean age at index, years (SD)**	56.81 (13.9)	54.06 (14.9)	0.190	56.81 (13.9)	56.93 (14.0)	0.009
**Female**	22,587 (81.1)	84,655 (76.2)	0.121	22,573 (81.1)	22,818 (82.0)	0.023
**Body mass index ≥ 30 kg/m²**	8,310 (29.8)	34,066 (30.6)	0.018	8,308 (29.8)	8,349 (30.0)	0.003
**Race**						
White	17,633 (63.3)	75,934 (68.3)	0.106	17,631 (63.4)	17,584 (63.2)	0.004
Black or African American	3,297 (11.8)	13,134 (11.8)	0.001	3,296 (11.8)	3,328 (12.0)	0.004
Asian	1,335 (4.8)	3,755 (3.4)	0.072	1,334 (4.8)	1,298 (4.7)	0.006
Other	836 (3.0)	4,037 (3.6)	0.035	836 (3.0)	827 (3.0)	0.002
**Risk factors**						
Tobacco use	413 (1.5)	2,013 (1.8)	0.026	413 (1.5)	338 (1.2)	0.023
Nicotine dependence	1,404 (5.0)	6,556 (5.9)	0.038	1,403 (5.0)	1,237 (4.4)	0.028
Alcohol-related disorders	231 (0.8)	1,276 (1.2)	0.032	231 (0.8)	173 (0.6)	0.025
**Comorbidities**						
Hypertension	7,446 (26.7)	26,630 (24.0)	0.064	7,438 (26.7)	7,342 (26.4)	0.008
Type 2 diabetes mellitus	3,099 (11.1)	10,909 (9.8)	0.043	3,095 (11.1)	2,976 (10.7)	0.014
Hyperlipidemia	3,941 (14.2)	13,236 (11.9)	0.067	3,935 (14.1)	3,727 (13.4)	0.022
Chronic kidney disease	1,335 (4.8)	3,879 (3.5)	0.065	1,328 (4.8)	1,252 (4.5)	0.013
COPD	1,334 (4.8)	3,902 (3.5)	0.064	1,330 (4.8)	1,175 (4.2)	0.027
Heart failure	1,085 (3.9)	2,346 (2.1)	0.105	1,076 (3.9)	986 (3.5)	0.017
Systemic lupus erythematosus	932 (3.4)	1,935 (1.7)	0.102	923 (3.3)	871 (3.1)	0.011
Psoriasis	1,565 (5.6)	6,831 (6.2)	0.022	1,565 (5.6)	1,436 (5.2)	0.021
**Medication**						
Betamethasone	820 (3.0)	2,615 (2.4)	0.037	816 (2.9)	743 (2.7)	0.016
Cortisone	38 (0.1)	110 (0.1)	0.011	38 (0.1)	43 (0.2)	0.005
Deflazacort	10 (0.04)	25 (0.02)	0.008	10 (0.04)	10 (0.04)	0.000
Dexamethasone	2,602 (9.3)	7,076 (6.4)	0.111	2,593 (9.3)	2,442 (8.8)	0.019
Hydrocortisone	1,061 (3.8)	3,233 (2.9)	0.050	1,057 (3.8)	972 (3.5)	0.016
Methylprednisolone	5,240 (18.8)	16,859 (15.2)	0.097	5,231 (18.8)	5,169 (18.6)	0.006
Prednisone	8,798 (31.6)	38,988 (35.1)	0.074	8,795 (31.6)	8,750 (31.4)	0.003
Prednisolone	1,512 (5.4)	3,323 (3.0)	0.122	1,502 (5.4)	1,504 (5.4)	0.000

COPD, chronic obstructive pulmonary disease; JAKi, Janus kinase inhibitor; SD, standard deviation; SMD, standardized mean difference; TNFi, tumor necrosis factor inhibitor. Data are presented as number (percentage) unless otherwise indicated.
