# Supplementary material for: Cryptococcosis After Initiation of Janus Kinase Inhibitors or Biologic DMARDs in Rheumatoid Arthritis: Three Pairwise Active-Comparator Analyses of Propensity Score-Matched TriNetX Cohorts, 2005–2025
Source: Pharmaceuticals (Basel). 2026 Oct 19;19(10):1650. doi: 10.3390/ph19101650 (PMC13708528; doi:10.3390/ph19101650)
Supplement: Supplementary file 1 [file pharmaceuticals-19-01650-s001.zip › Supplementary Table S3.pdf]

Supplementary Table S3. Baseline characteristics before and after propensity score matching: JAKi versus non-TNF bDMARDs.

Variable	Before matching			After matching		
	**JAKi** (n = 33,287)	**Non-TNF bDMARDs** (n = 48,734)	**SMD**	**JAKi** (n = 32,440)	**Non-TNF bDMARDs** (n = 32,440)	**SMD**
**Mean age at index, years (SD)**	56.06 (13.9)	57.17 (14.8)	0.077	56.18 (13.9)	55.98 (14.4)	0.014
**Female**	26,770 (80.4)	39,260 (80.6)	0.003	26,116 (80.5)	26,223 (80.8)	0.008
**Body mass index ≥ 30 kg/m²**	10,667 (32.0)	15,258 (31.3)	0.016	10,238 (31.6)	10,107 (31.2)	0.009
**Race**						
White	21,492 (64.6)	30,950 (63.5)	0.022	20,835 (64.2)	20,897 (64.4)	0.004
Black or African American	3,872 (11.6)	5,951 (12.2)	0.018	3,806 (11.7)	3,760 (11.6)	0.004
Asian	1,558 (4.7)	2,020 (4.2)	0.026	1,533 (4.7)	1,491 (4.6)	0.006
Other	969 (2.9)	1,964 (4.0)	0.061	965 (3.0)	1,006 (3.1)	0.007
**Risk factors**						
Tobacco use	490 (1.5)	842 (1.7)	0.020	485 (1.5)	405 (1.2)	0.021
Nicotine dependence	1,804 (5.4)	2,702 (5.5)	0.005	1,743 (5.4)	1,595 (4.9)	0.021
Alcohol-related disorders	295 (0.9)	452 (0.9)	0.004	286 (0.9)	266 (0.8)	0.007
**Comorbidities**						
Hypertension	8,789 (26.4)	14,338 (29.4)	0.067	8,545 (26.3)	8,269 (25.5)	0.019
Type 2 diabetes mellitus	3,629 (10.9)	5,935 (12.2)	0.040	3,521 (10.8)	3,242 (10.0)	0.028
Hyperlipidemia	4,575 (13.7)	7,270 (14.9)	0.033	4,441 (13.7)	4,169 (12.8)	0.025
Chronic kidney disease	1,483 (4.5)	3,184 (6.5)	0.091	1,468 (4.5)	1,394 (4.3)	0.011
COPD	1,519 (4.6)	2,532 (5.2)	0.029	1,476 (4.6)	1,374 (4.2)	0.015
Heart failure	1,142 (3.4)	2,836 (5.8)	0.114	1,138 (3.5)	1,076 (3.3)	0.011
Systemic lupus erythematosus	901 (2.7)	2,796 (5.7)	0.151	901 (2.8)	890 (2.7)	0.002
Psoriasis	2,277 (6.8)	1,464 (3.0)	0.178	1,432 (4.4)	1,410 (4.4)	0.003
**Medication**						
Betamethasone	1,056 (3.2)	1,310 (2.7)	0.029	975 (3.0)	880 (2.7)	0.018
Cortisone	34 (0.1)	101 (0.2)	0.027	34 (0.1)	27 (0.1)	0.007
Deflazacort	10 (0.03)	32 (0.07)	0.016	10 (0.03)	10 (0.03)	0.000
Dexamethasone	3,159 (9.5)	4,499 (9.2)	0.009	3,027 (9.3)	2,843 (8.8)	0.020
Hydrocortisone	1,318 (4.0)	2,679 (5.5)	0.072	1,287 (4.0)	1,175 (3.6)	0.018
Methylprednisolone	6,552 (19.7)	10,651 (21.9)	0.054	6,347 (19.6)	6,238 (19.2)	0.008
Prednisone	11,726 (35.2)	19,989 (41.0)	0.119	11,450 (35.3)	11,612 (35.8)	0.010
Prednisolone	1,719 (5.2)	2,807 (5.8)	0.026	1,694 (5.2)	1,690 (5.2)	0.001

COPD, chronic obstructive pulmonary disease; JAKi, Janus kinase inhibitor; non-TNF bDMARDs, non-tumor necrosis factor biologic disease-modifying antirheumatic drugs; SD, standard deviation; SMD, standardized mean difference. Data are presented as number (percentage) unless otherwise indicated.
